# Supplementary figures and images for: A Cre-driver rat model for anatomical and functional analysis of glucagon (Gcg)-expressing cells in the brain and periphery
Source: Mol Metab. 2022 Nov 8;66:101631. doi: 10.1016/j.molmet.2022.101631 (PMC9677222; doi:10.1016/j.molmet.2022.101631)

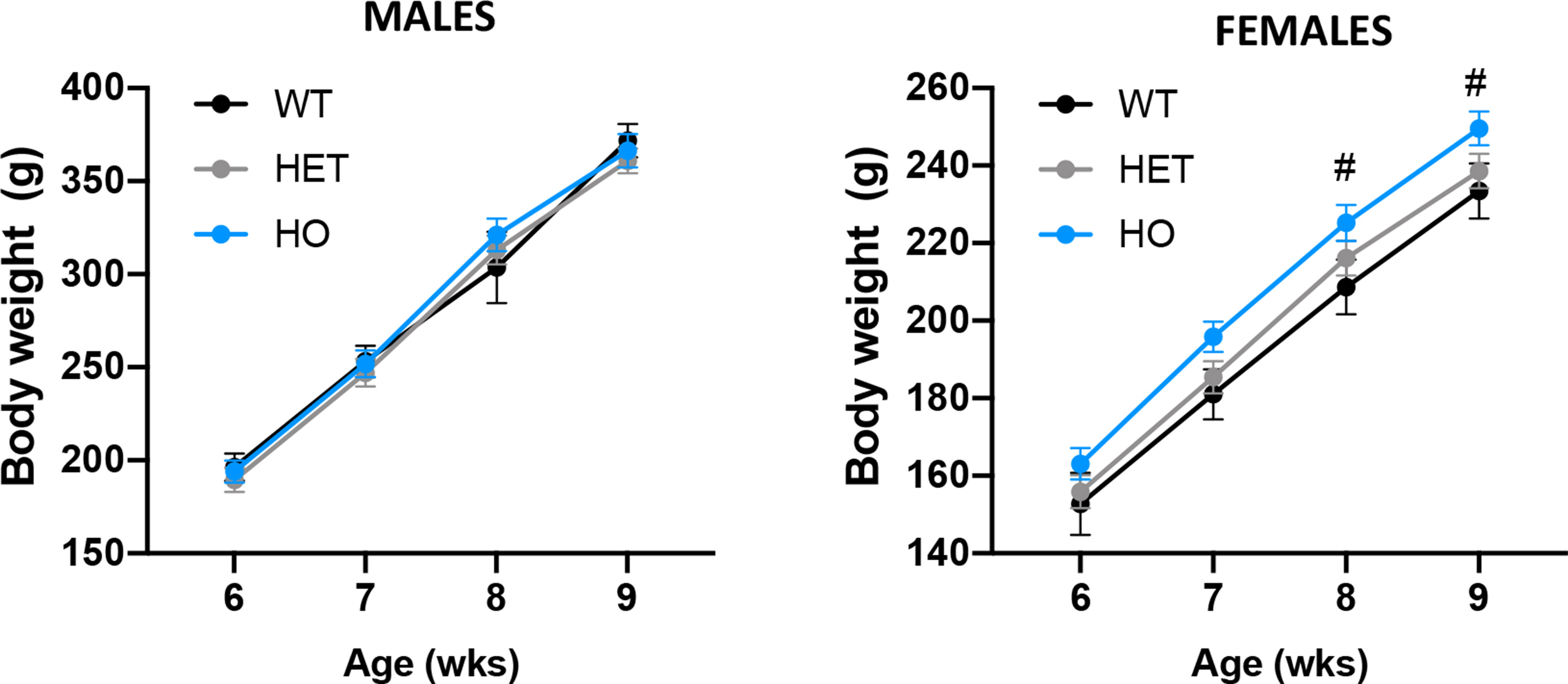

Supplement: Supplementary Figure 1 — Body weight (BW) in young Gcg-Cre rats bred in the Janvier facility. Left, Male Gcg-Cre rats display similar growth curves and BW from weeks 6–9 postnatal, regardless of genotype. Right, Female Homo Gcg-Cre rats display slightly higher BW compared to WT rats at weeks 8 and 9, but the difference was not significant (#P < 0.1 and >0.05). [file figs1.jpg]

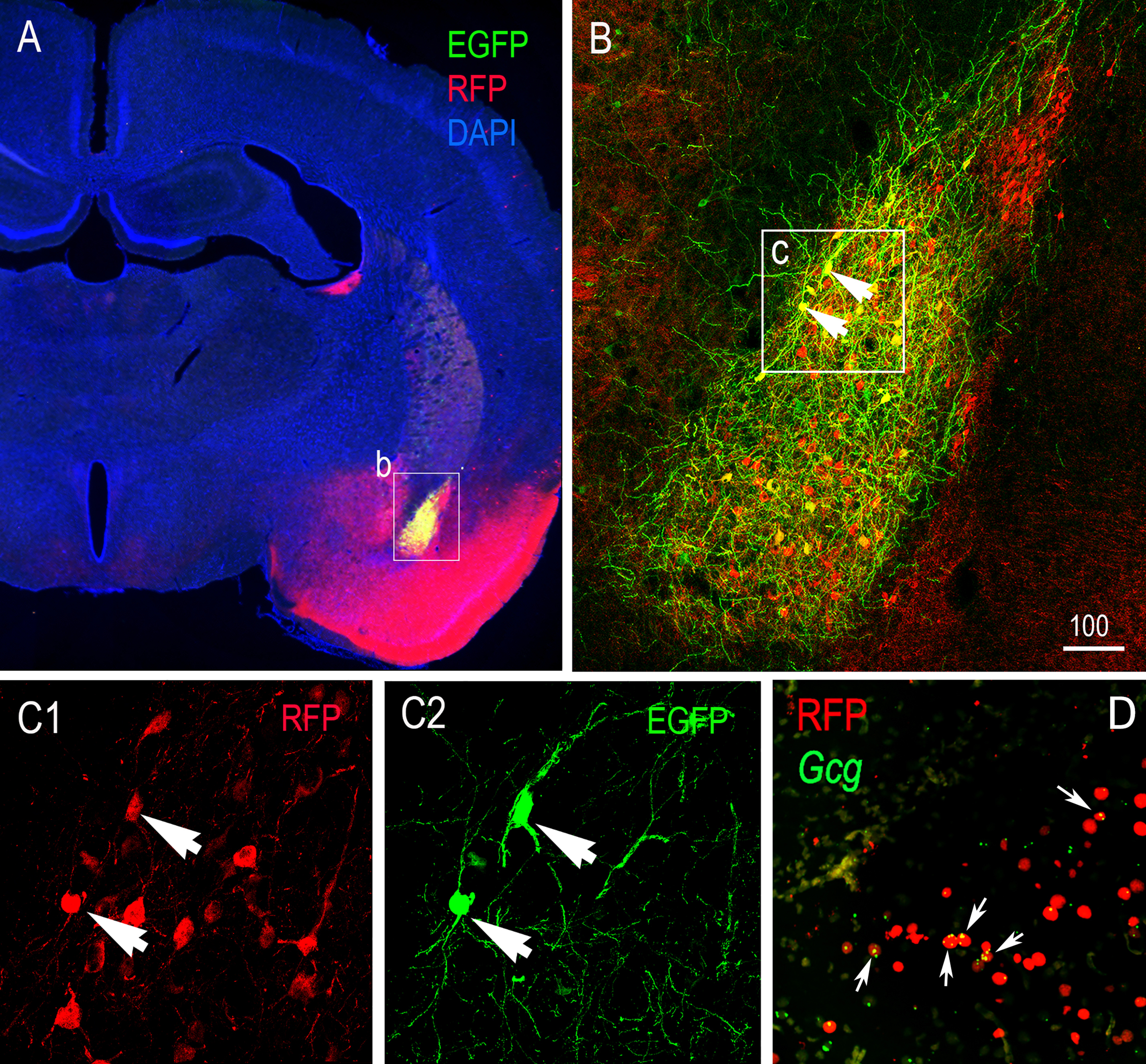

Supplement: Supplementary Figure 2 — Panels A-C2 depict epifluorescent (A) and confocal (B, C1, C2) images of EGFP viral labeling (green) in an adult female Gcg-Cre/tdTom reporter rat after microinjection of cre-dependent AAV expressing EGFP reporter. A, BLA injection site (b, boxed inset shown at higher magnification in panel B). Red (RFP) labeling is immunofluorescently enhanced tdTom reporter labeling. Blue DAPI counterstain. B, EGFP-expressing BLA neurons give rise to extensive dendritic and axonal processes. In the boxed inset (c), white arrows point out two neurons that are double-labeled for RFP (tdTom reporter) and EGFP. Boxed inset is shown at higher magnification in C1 (RFP channel) and C2 (EGFP channel). Many RFP-positive neurons do not express EGFP viral reporter. D, RNAscope FISH reveals Gcg mRNA expression in a subset of BLA tdTom (RFP)-positive neurons in a neonatal (P10) Gcg-Cre/tdTom reporter rat. White arrows point out double-labeled cells. Scale bar in B is in microns. [file figs2.jpg]

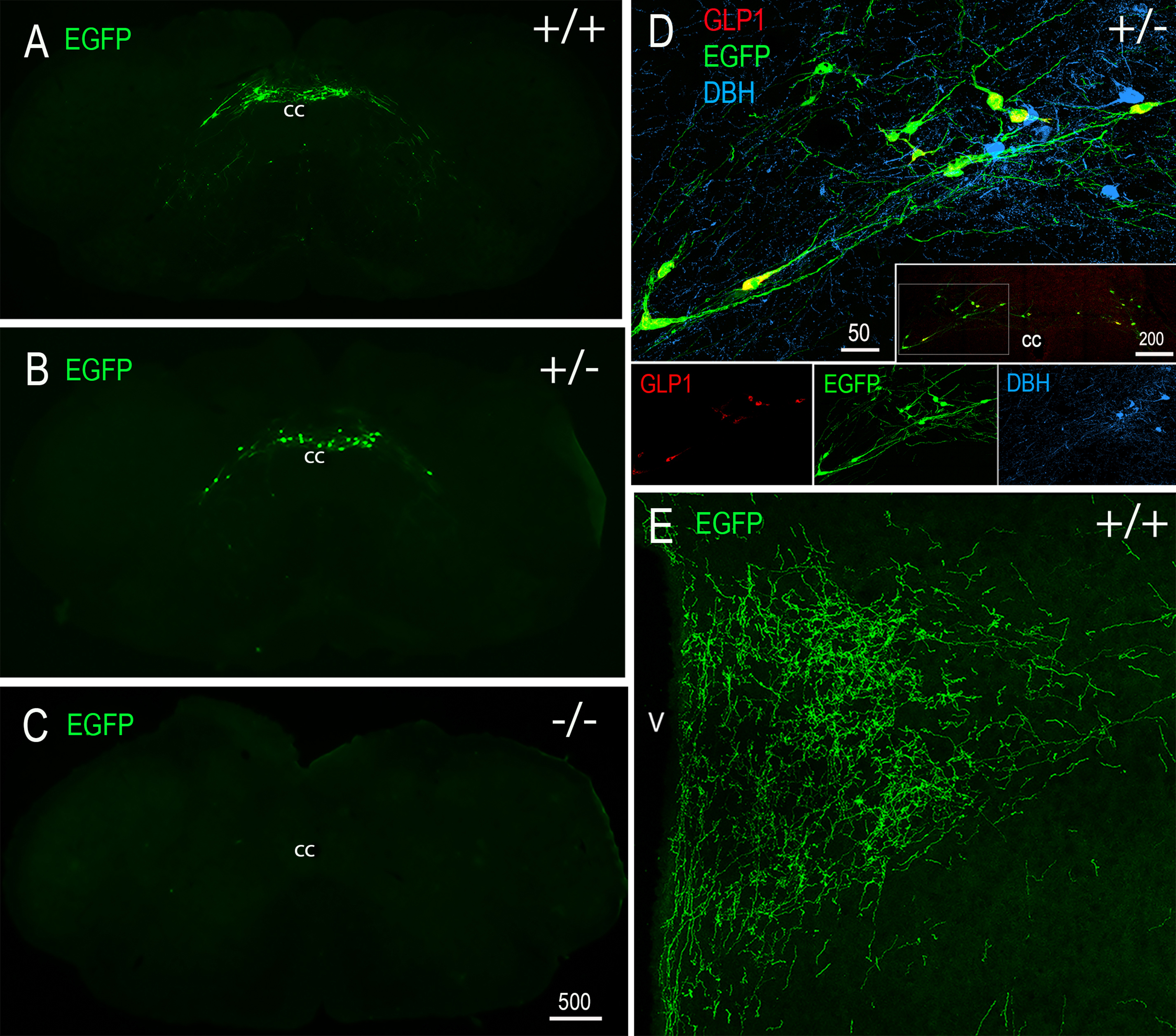

Supplement: Supplementary Figure 3 — Cre-dependent EGFP reporter labeling in adult Homo (A, +/+), Het (B, −/−), and WT (C, −/−) Gcg-Cre rats after cNTS-targeted AAV injections. No reporter labeling is observed in WT rats, which lack iCre expression. cc, central canal.D, inset shows boxed region enlarged in panel D, from a Het Gcg-Cre rat. All EGFP-positive cells are GLP1-positive, and vice versa, as shown in the 3 smaller panels below D. DBH-positive noradrenergic neurons are not transfected by the Cre-dependent AAV. E, EGFP-positive axons within the paraventricular nucleus of the hypothalamus in a Homo rat that received cNTS-targeted AAV injection. V, 3rd ventricle. Scale bars are in microns. [file figs3.jpg]

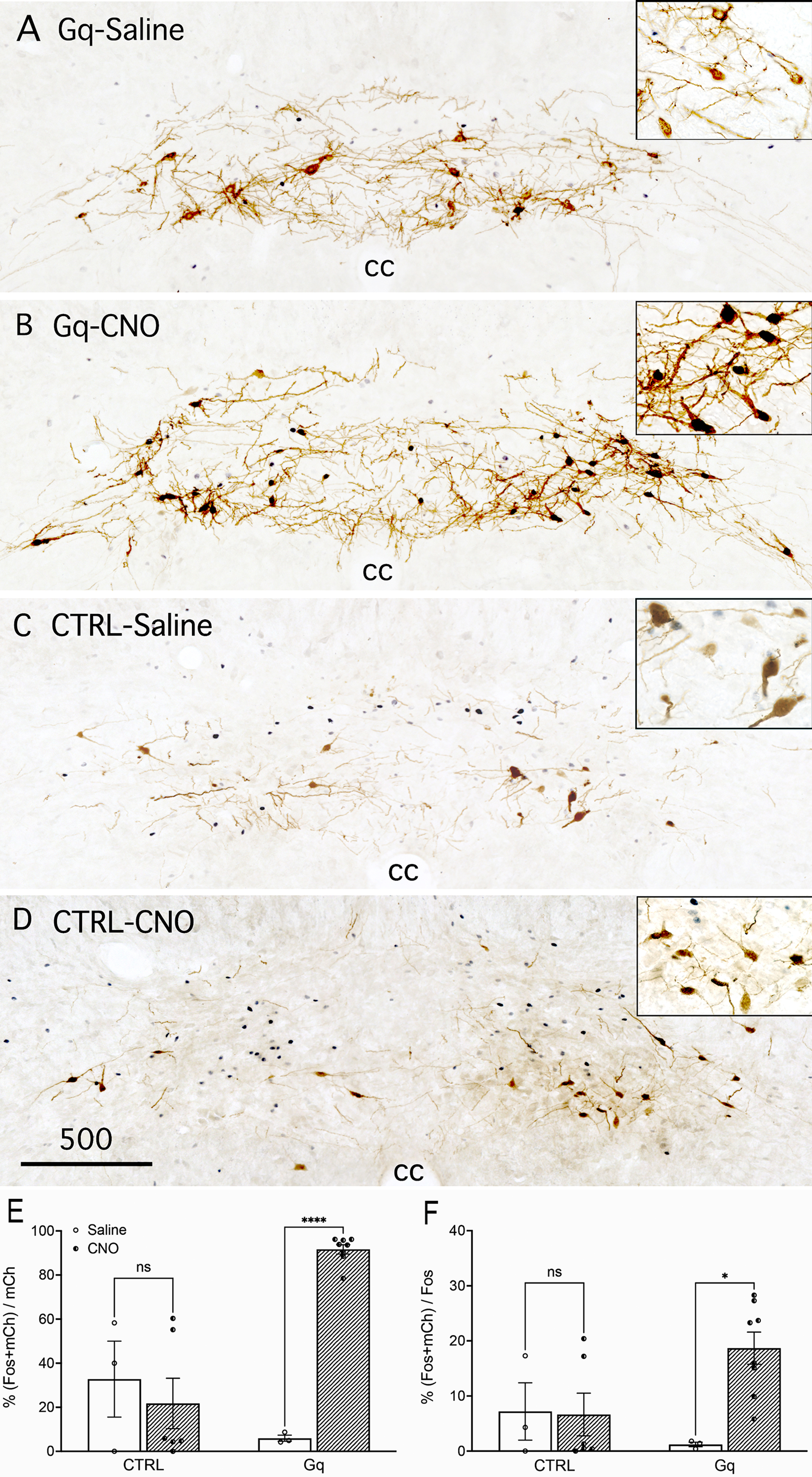

Supplement: Supplementary Figure 4 — Confirmation of GqDREADD expression and CNO-induced activation of cNTS neurons in adult female Het Gcg-Cre rats. Tissue sections were double-labeled for immunoperoxidase localization of nuclear cFos (blue/black) and mCherry cytoplasmic reporter (brown). Rats received cNTS-targeted injections of Cre-dependent AAV expressing GqDREADD (Gq) or control AAV (CTRL) expressing only mCherry. Rats were injected i.p. with either saline vehicle or CNO (1 mg/kg BW) 90 min before perfusion. A, GqDREADD-expressing cNTS neurons are not activated to express cFos after i.p. saline. B, GqDREADD-expressing cNTS neurons are activated after i.p. injection of CNO. C, Control virus mCherry-labeled cells are not activated to express cFos after i.p. saline injection. D, Control virus mCherry-labeled cells are not activated to express cFos after i.p. CNO. E, summary data quantifying the proportion of mCherry-positive transfected neurons expressing cFos in rats injected with control virus (CTRL) or with GqDREADD-expressing virus (Gq). CNO does not increase activation in CTRL rats, but markedly increases activation in Gq rats (∗∗P < 0.001). F, summary data quantifying the proportion of cFos-positive cNTS neurons that are mCherry labeled. CNO does not increase this proportion in CTRL rats, but significantly increases this proportion in Gq rats (∗P < 0.05). [file figs4.jpg]

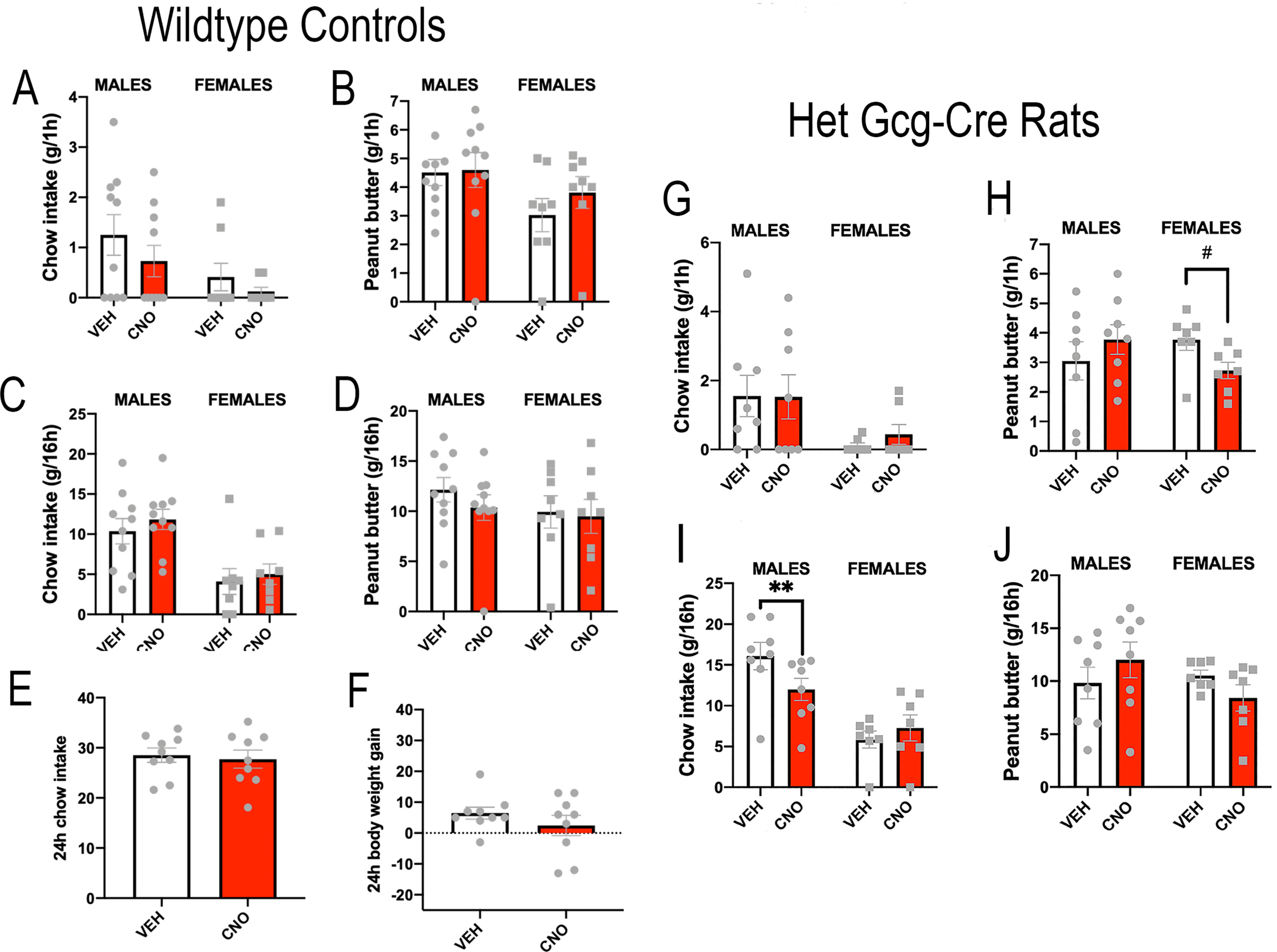

Supplement: Supplementary Figure 5 — Left, data from wildtype control (−/−) rats. Chow vs. peanut butter intake (A-D, 2-food choice test, E, chow only) in male and female WT Gcg-Cre rats with cNTS AAV injections of Cre-dependent GqDREADDs, followed by intra-cNTS administration of CNO (red bars) or vehicle (open bars) before dark-onset food access. A, B, 1 h intake of chow (A) or peanut butter (B) does not differ in either sex following vehicle vs. CNO treatment. C, D, 16 h overnight intake of chow (C) or peanut butter (D) does not differ in either sex following vehicle vs. CNO treatment. E, 24 h chow intake (no choice) does not differ in male rats after vehicle vs. CNO treatment. F, body weight gain does not differ in male rats after vehicle vs. CNO treatment. Right, data from Het (+/−) Gcg-Cre rats. Chow vs. peanut butter intake (2-food choice test) in male and female Het Gcg-Cre rats with cNTS AAV injections of Cre-dependent GqDREADDs, followed by intra-cNTS administration of vehicle (open bars) or CNO (red bars) before dark-onset food access. G, 1 h dark-onset chow intake; H, 1 h dark-onset peanut butter intake; I, 16 h overnight chow intake; J, 16 h overnight peanut butter intake. At the 1 h timepoint, CNO did not inhibit either chow (G) or peanut butter (H) intake in either sex compared to intake after vehicle treatment, although a strong trend towards inhibition of peanut butter intake was evident in females (panel H, #P = 0.05). At the 16 h timepoint, CNO significantly inhibited chow intake in male rats (panel I, ∗∗P < 0.05) but not in females. Peanut butter intake at 16 h (J) did not differ in either sex after vehicle vs. CNO treatment. [file figs5.jpg]
